# Synthesis and characterization of amine-functionalized graphene as a nitric oxide-generating coating for vascular stents

**DOI:** 10.1063/5.0192379

**Published:** 2024-09-24

**Authors:** Tanveer A. Tabish, Mian Zahid Hussain, Yangzhi Zhu, Jiabao Xu, Wei E. Huang, Marina Diotallevi, Roger J. Narayan, Mark J. Crabtree, Ali Khademhosseini, Paul G. Winyard, Craig A. Lygate

**Affiliations:** 1 Division of Cardiovascular Medicine, Radcliffe Department of Medicine, British Heart Foundation (BHF) Centre of Research Excellence, University of Oxford, Headington, Oxford OX3 7BN, United Kingdom; 2 School of Natural Sciences, Department of Chemistry, Chair of Inorganic and Metal-Organic Chemistry, Technical University of Munich (TUM), Lichtenbergstraße 4, 85748 Garching, Germany; 3 Terasaki Institute for Biomedical Innovation, Los Angeles, California 90064, USA; 4 Department of Engineering Science, University of Oxford, Oxford OX1 3PJ, United Kingdom; 5 Division of Biomedical Engineering, James Watt School of Engineering, University of Glasgow, Glasgow G12 8LT, United Kingdom; 6 Joint Department of Biomedical Engineering, University of North Carolina and North Carolina State University, Raleigh, North Carolina 27599, USA; 7 Department of Biochemical Sciences, School of Biosciences and Medicine, University of Surrey, Guildford GU2 7XH, United Kingdom; 8 University of Exeter Medical School, College of Medicine and Health, University of Exeter, Exeter EX1 2LU, United Kingdom; 9 School of Cardiovascular and Metabolic Health, University of Glasgow, Glasgow, G12 8TA, United Kingdom

## Abstract

Drug-eluting stents are commonly utilized for the treatment of coronary artery disease, where they maintain vessel patency and prevent restenosis. However, problems with prolonged vascular healing, late thrombosis, and neoatherosclerosis persist; these could potentially be addressed via the local generation of nitric oxide (NO) from endogenous substrates. Herein, we develop amine-functionalized graphene as a NO-generating coating on polylactic acid (PLA)-based bioresorbable stent materials. A novel catalyst was synthesized consisting of polyethyleneimine and polyethylene glycol bonded to graphene oxide (PEI-PEG@GO), with physicochemical characterization using x-ray diffraction, Raman spectroscopy, Fourier transform infrared spectroscopy, and thermogravimetric analysis. In the presence of 10 *μ*M S-nitrosoglutathione (GSNO) or S-nitroso-N-acetylpenicillamine (SNAP), PEI-PEG@GO catalyzed the generation of 62% and 91% of the available NO, respectively. Furthermore, PEI-PEG@GO enhanced and prolonged real-time NO generation from GSNO and SNAP under physiological conditions. The uniform coating of PEI-PEG@GO onto stent material is demonstrated via an optimized simple dip-coating method. The coated PLA maintains good biodegradability under accelerated degradation testing, while the PEI-PEG@GO coating remains largely intact. Finally, the stability of the coating was demonstrated at room temperature over 60 days. In conclusion, the innovative conjugation of polymeric amines with graphene can catalyze the generation of NO from *S*-nitrosothiols at physiologically relevant concentrations. This approach paves the way for the development of controlled NO-generating coatings on bioresorbable stents in order to improve outcomes in coronary artery disease.

## Introduction

Coronary artery disease is characterized by the accumulation of atherosclerotic plaque, which causes the blood vessels supplying the heart to gradually become occluded and accounts for an estimated 17.9 × 10^6^ deaths annually, or 31% of all global deaths.^1^ The standard treatment consists of angioplasty to clear the blockage, followed by implantation of a stent, an expandable tubular mesh or scaffold, which keeps the blood vessel patent during the healing process.^2^ Originally, stents were made from bare metal (BMS), but these were associated with a high incidence of restenosis, a re-narrowing of the injured artery, driven by damaged endothelial cells, uncontrolled smooth muscle cell (SMC) proliferation, and a persistent inflammatory response.^3^ This situation led to the introduction of drug-eluting stents (DES), whereby a metal stent is coated with biodegradable polymers for the local delivery of antiproliferative drugs (e.g., paclitaxel or sirolimus), which reduce the likelihood of restenosis and have become the gold standard in percutaneous coronary intervention.^4^ However, the use of antiproliferative drugs can delay the growth and migration of endothelial cells (ECs) (re-endothelialization), thereby prolonging chronic inflammation and the vascular healing process, which in turn increases the incidence of late-onset clotting (thrombosis) and neoatherosclerosis.^4^ As such, an ideal stent should exhibit anticorrosive, smooth, flexible, low friction, anti-thrombogenic, and biocompatible characteristics. It should also release compounds that inhibit SMC proliferation while simultaneously promoting EC survival, preventing thrombosis, and dampening the inflammatory response.

Normal healthy vascular ECs continuously generate nitric oxide (NO) via the enzymatic conversion of L-arginine by NO synthase (NOS).^5–7^ NO has many crucial roles in maintaining vascular homeostasis; e.g., it is an important vasodilator and inhibits the activation of platelets and inflammatory cells to prevent thrombosis and local inflammatory responses. Furthermore, it promotes EC survival and inhibits SMC proliferation;^8^ hence, a NO-eluting stent would theoretically realize all of the ideal properties required to promote vascular healing and prevent restenosis. To this end, several approaches have been investigated to provide controlled and sustained NO delivery.^9^ Non-catalytic approaches involve the attachment of exogenous NO donors such as NO gas, diazeniumdiolates (NONOate), and *S*-nitrosothiols (RSNO), but are *de facto* constrained by the limited pool of conjugated NO donors to draw upon. Moreover, the short therapeutic half-lives of many NO donors limit their use for long-term vascular implants.^10^ In the alternative, catalytic approach, materials are engineered to decompose endogenous NO sources into NO [e.g., RSNOs, such as *S*-nitrosoglutathione (GSNO), *S*-nitrosocysteine, and *S*-nitrosoalbumin that are naturally replenished *in vivo*]. As such, catalyst materials, e.g., Cu^2+^, ascorbic acid, and transition metal ions, may provide the advantage of continuous and localized production of NO and show superior catalytic activity by tailoring the appropriate ratios and concentrations of both the catalyst materials and NO sources to achieve physiologically relevant amounts of NO.^11,12^

Three classes of catalysts have been reported in the literature to generate NO from endogenous sources. First, metallic ions, such as ions of mercury (Hg^2+^),^13^ gold (Au^+^),^14^ and particularly copper (Cu^2+^),^15,16^ have been reported to decompose RSNO into NO. For example, the glutamate residue in GSNO binds with Cu^2+^ ions to facilitate NO production; however, this reaction is impeded by the accumulation of oxidized glutathione (GSSG), indicating that the catalytic capacity of copper ions is negatively influenced by the presence of disulfide bonds.^17^ Furthermore, copper ion leaching results in decreased NO generation by up to 50% over 15 days.^18,19^ The second class of catalyst includes group 16 elements, including selenium^20^ and tellurium;^21^ e.g., organoselenium immobilized to PEI has been shown to effectively catalyze the decomposition of GSNO to NO in the presence of free thiols such as glutathione;^22^ however, leaching of selenium and deactivation of the catalyst are potential limitations. The third class is small nucleophiles, such as hydrogen peroxide,^23,24^ hydrazine^25^ (undesirable due to potential toxicity), and ascorbic acid,^26^ which can attack the electrophilic nitroso groups of RSNO.

Herein, we explore a novel catalytic NO-generating strategy using functionalized graphene oxide (GO). GO possesses a number of desirable properties, including high hydrophilicity, excellent stability in solvents and biological buffers, and ease of functionalization owing to the existence of oxygen-containing functional groups on its surface (e.g., carboxyl, hydroxyl, and epoxide), which allow it to be tailored as a highly efficient carrier for drugs and biomolecules.^27,28^ In a biological environment, the nitroso groups of GSNO and RSNO can undergo electrophilic attack by nucleophiles, leading to the release of NO as a leaving group.^29^ Polymers containing amino groups, such as PEI, represent one of the most versatile nucleophiles and have been widely investigated for drug delivery.^30,31^ We, therefore, hypothesize that PEI-conjugated GO could act as a catalyst to decompose *S*-nitrosothiols to generate therapeutic amounts of NO.

Further chemical functionalization of GO with polymers such as polyethylene glycol (PEG) offers superior solubility, stability, and improved biocompatibility.^32^ PEG is considered bioinert and is clinically approved for drug delivery due to its excellent biocompatibility and its ability to reduce nonspecific effects of protein adsorption and colloidal aggregation.^33^ PEGylation of GO enhances its capacity to carry and release therapeutic agents in a controlled manner. Additionally, the inherent mechanical strength of GO combined with the mechanical flexibility of PEG make it an ideal candidate for coating stents, which require materials that can withstand the mechanical stresses of implantation and the dynamic environment of blood vessels while maintaining structural integrity.

In this work, we report the novel design and synthesis of a NO-generating stent coating by combining the functionalization of GO with PEI and PEG (PEI-PEG@GO). Physicochemical characterization involved assessment using Fourier transform infrared (FTIR) spectroscopy, Raman spectroscopy, and scanning electron microscopy (SEM); we demonstrate that this unique PEI-PEG@GO combination facilitates the persistent catalytic generation of NO from *S*-nitrosothiols. We show that PEI-PEG@GO can be used to uniformly coat three-dimensional (3D)-printed bioresorbable stent material [polylactic acid (PLA)] via a simple dip-coating method without grossly affecting biodegradability. To the best of our knowledge, this is the first study reporting NO generation via the reaction between amine-functionalized GO and nitroso groups. Our findings represent a first step toward the design of 3D-printed stents made of bioresorbable polymers with intrinsic NO-generating capabilities to promote vascular healing and prevent restenosis.

## Results and Discussion

### Design, synthesis, and optimization of amine-functionalized graphene

**FIG. 1:**
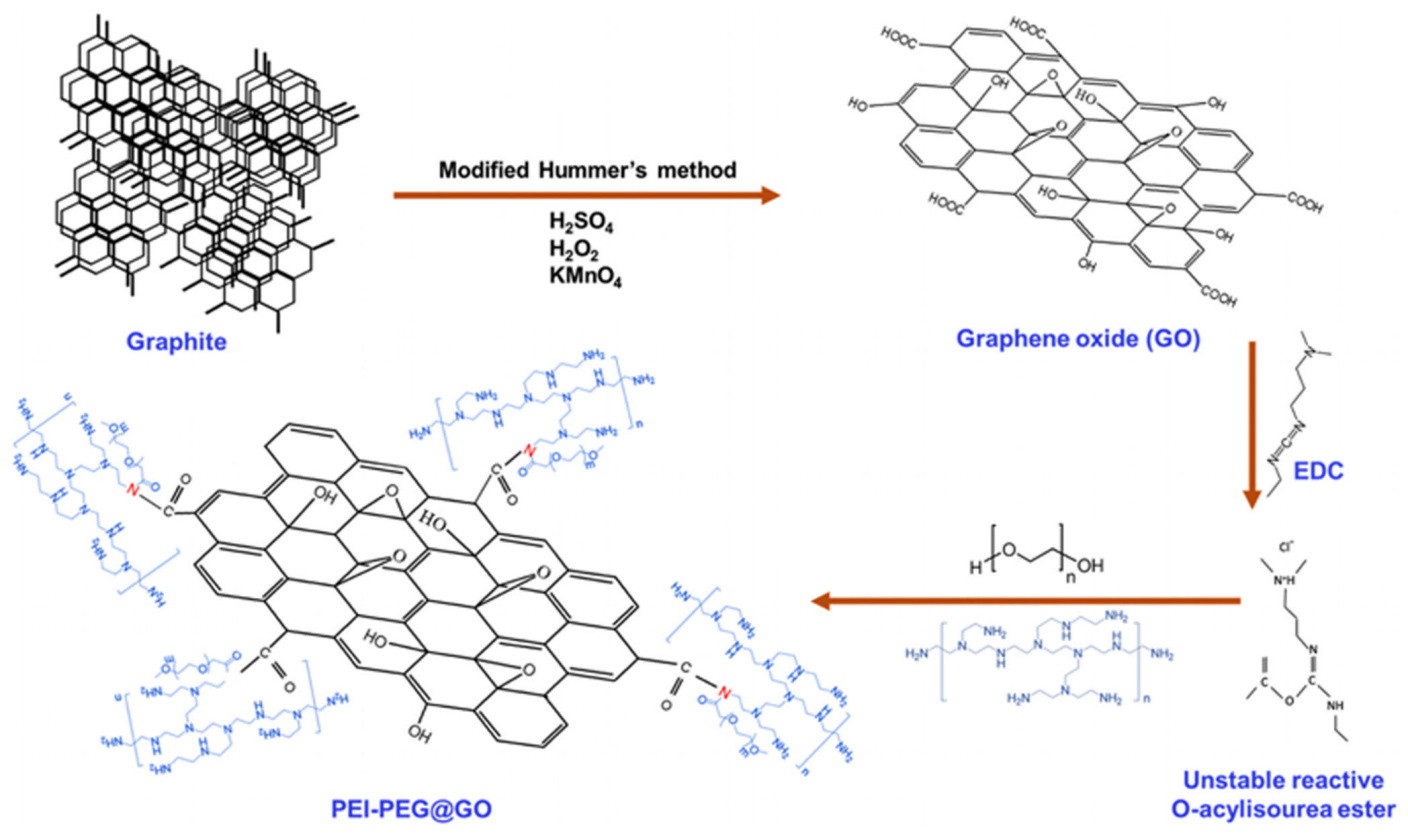
A schematic representation of the design and synthesis of functionalized GO conjugate (termed as PEI-PEG@GO). First, GO was synthesized by oxidizing graphite following the modified Hummer’s method. GO was functionalized with PEI and PEG by the formation of an amide bond between PEI, PEG, and GO in the presence of EDC. The product was washed 3–4 times with an aqueous solution that contained 10% NaCl in order to remove any unreacted PEI, PEG, and urea.

The detailed synthesis and characterization of GO is described in our previous work.^34,35^ PEG and PEI were covalently conjugated to GO by the formation of amide bonds between PEI, PEG, and GO using N-(3-Dimethylaminopropyl)-N′-ethylcarbodiimide hydrochloride (EDC). EDC coupling involves the conjugation of carboxylic acid functional groups to primary amines. GO possesses carboxylic acid functional groups on its surface, which act as anchoring agents for the functionalization of GO with biomolecules through electrostatic interactions. EDC in the presence of carboxylic groups forms O-acylisourea, which then reacts with primary amino groups to form an amide bond. This reaction results in a by-product of urea derivatives, which are purified using centrifugation or filtration methods.^36^ The synthesis has also been schematically illustrated in Fig. 1. GO has good dispersibility in water without significant aggregation; however, the attachment of hydrophilic polymers such as PEG significantly improves the dispersibility and stability of GO in biological buffers, which is crucial for biomedical applications.^32^ Moreover, PEI was chosen to introduce primary amine groups on the surface of GO. PEI contains a significant proportion of amino groups, which may serve as building blocks for the catalytic decomposition of endogenous NO sources; however, PEI tends to aggregate over time and becomes unstable.^37^ The instability of PEI@GO in serum has been attributed to protein adsorption on the surface of GO by electrostatic attractions,^38–40^ thereby neutralizing the surface charge and resulting in GO aggregation. The addition of a hydrophilic protective layer of PEG should prevent undesirable binding of proteins to the negatively charged surface of GO.^32^ It has previously been shown that PEG significantly improves the stability of PEI conjugated with a diverse range of materials and biomolecules;^41–43^ therefore, we prepared both PEI@GO and PEI-PEG@GO.

### Physicochemical characterization

**FIG. 2:**
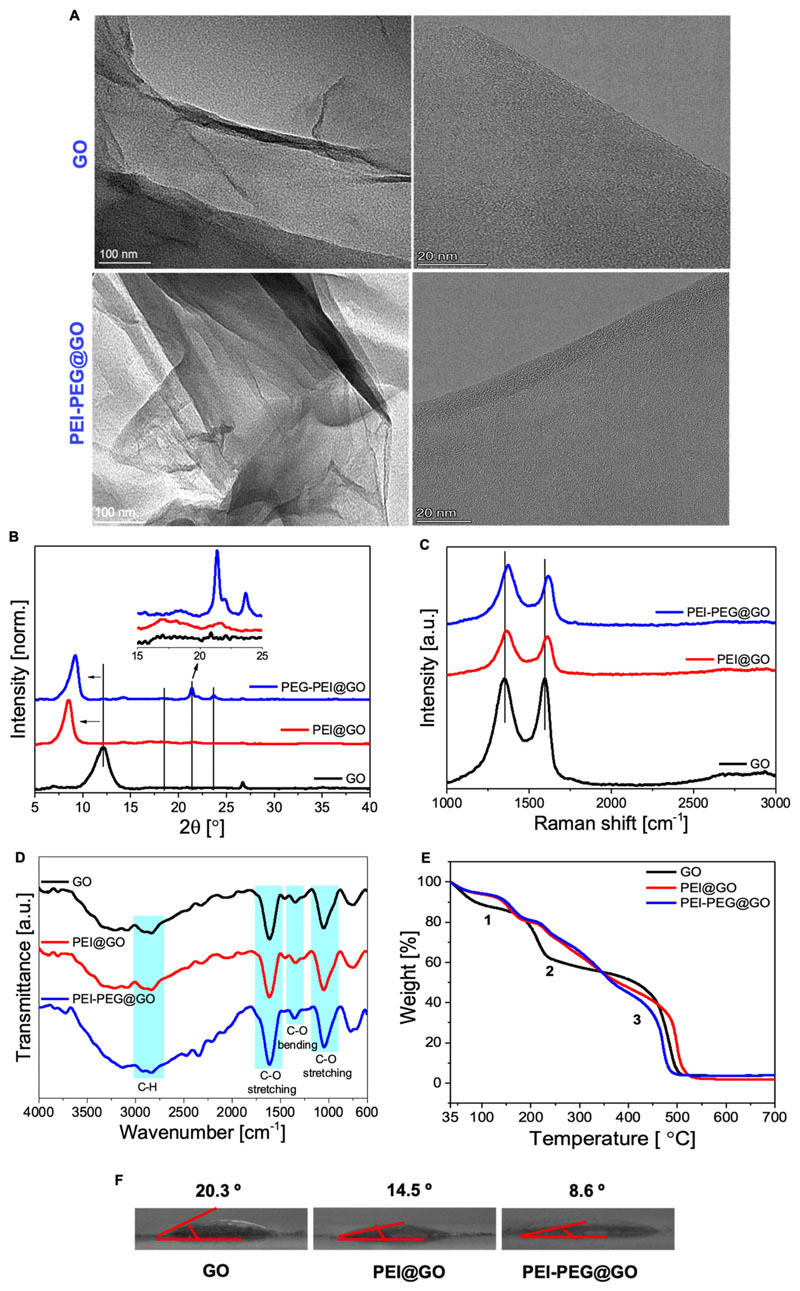
Basic characterization of GO, PEI@GO, and PEI-PEG@GO. (a) Transmission electron microscopy (TEM) images, (b) XRD patterns, (c) Raman spectroscopy, (d) FTIR spectra, (e) thermogravimetric analysis (TGA), and (f) water contact angle measurements.

The morphology of GO and PEI-PEG@GO was evaluated using TEM, with GO exhibiting a distribution of stacked layers in a flat manner. In contrast, the PEI-PEG@GO showed a distribution of stacked layers in an irregular manner due to the amide linkage [Fig. 2(a)]. This demonstrates that the conjugation of PEI and PEG did not modify the stacked layered-like morphology of GO. X ray diffraction (XRD) analysis showed the characteristic sharp diffraction peak of GO at 2*θ* = 12.5° corresponding to the (001) plane and indicating the presence of functional groups in the basal planes of graphene [Fig. 2(b)]. This diffraction peak was shifted to a low angle of 7.5° in both PEI@GO and PEI-PEG@GO due to the incorporation of PEI and PEG within the graphene, which increases the distance between layers. The functionalized PEI@GO showed a broadening of the diffraction peak at 2*θ* =23° indicating the introduction of amorphous structure to GO, whereas the appearance of a new peak at 21.5° in PEI-PEG@GO reveals the attachment of amorphous polymer.

Raman spectroscopy identified the typical Raman bands of graphene [Fig. 2(c)], with the D band at 1350 cm^−1^ representing the breathing mode of aromatic rings and the introduction of other conjugated molecules, while the G band at 1600 cm^−1^ is assigned to the graphitic nature representing the first-order scattering of carbon sp^2^ atoms.^44^ The reduction in both D and G bands indicates the molecular charge transfer resulting from the covalent conjugation of GO with PEI and PEG. The functionalization interaction of PEI-PEG@GO was further characterized by FTIR, where spectra show the presence of −CH (~2900 cm^−1^), −COOH (~1717 cm^−1^), and C=O (~1110cm^−1^) functional groups in GO [Fig. 2(d)]. The peak at ~1400 cm^−1^ is assigned to the stretching vibrations of the C=O band of carbonyl groups, indicating the lack of carboxyl groups on the surface of GO, while the appearance of this peak in the functionalized sample is attributed to the ester bonding between PEI, PEG, and GO. The FTIR spectra of functionalized samples revealed that all the GO peaks were preserved with a slight shift in peak positions and variation in intensity, confirming the successful conjugation of GO with PEI and PEG.

The relative amounts of PEI and PEG on the surface of GO were further estimated by thermogravimetric analysis [TGA; Fig. 2(e)]. Three obvious peaks at ~100, 230, and 500 °C (labeled as 1, 2, and 3) are attributed to the evaporation of residual water between sheets, the elimination of oxygen-containing functional groups, and the breakdown and combustion of carbon, respectively.^45^ In particular, the PEI@GO and PEI-PEG@GO samples exhibited more pronounced weight loss than GO between 250 and 400 °C, which can be attributed to the decomposition of covalently bonded PEI and PEI-PEG molecules.^45,46^ The proportion of sample weight for PEI and PEG on PEI-PEG@GO is calculated to be 15% and 4.2%, respectively. Water contact angle profiles of GO, PEI@GO, and PEI-PEG@GO gave values of 20.3°, 14.5°, and 8.6°, respectively [Fig. 2(f)], indicating the solubility of functionalized GO was better than GO alone and represents further indirect evidence for the successful conjugation of PEI-PEG with GO.

### Catalytic generation of NO

**FIG. 3:**
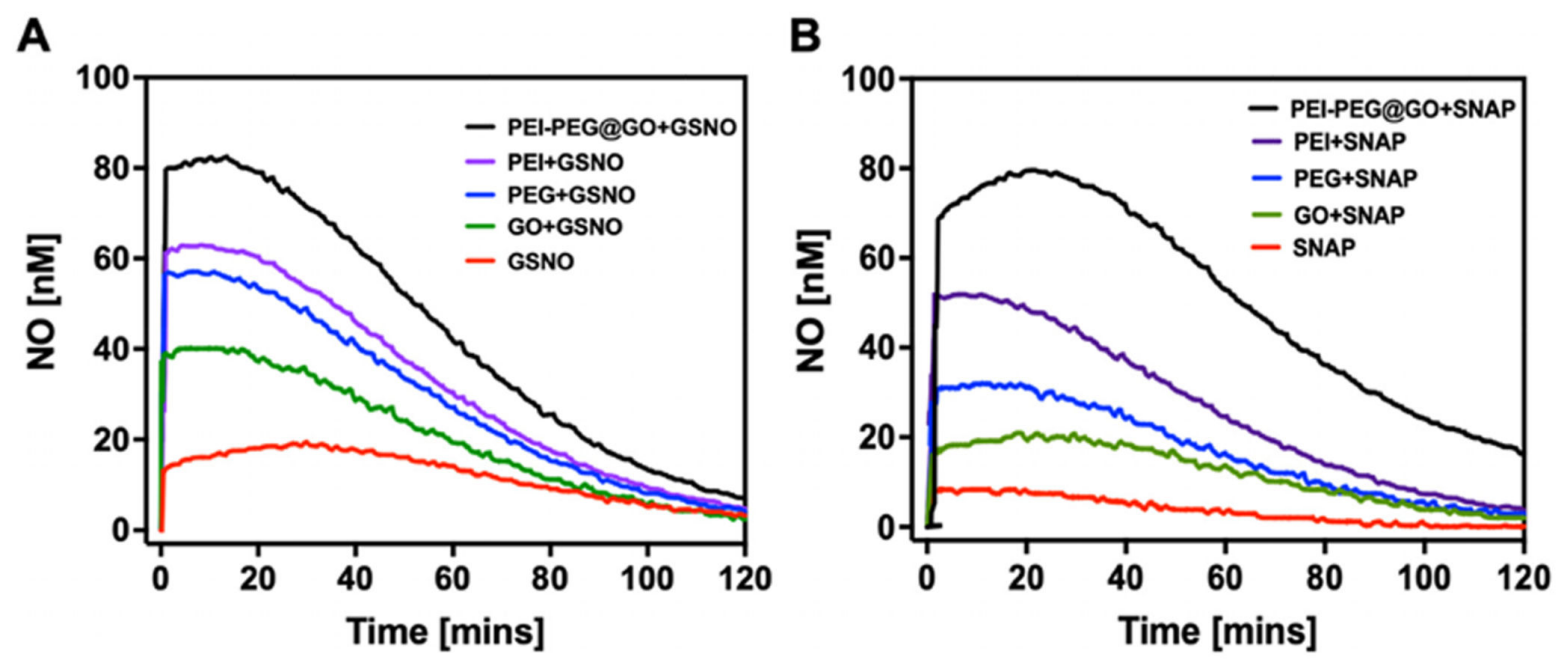
Quantification of NO release from GSNO and SNAP with and without different formulations using a NO electrode sensor under physiological conditions. (a) NO release measurements from GSNO alone (10 *μ*M) and GSNO (10 *μ*M) in the presence of different formulations of GO, PEI, PEG, and PEI-PEG@GO at the concentration of 250 *μ*g/ml (n = 3 independent samples). (b) NO release measurements from SNAP alone (10 *μ*M) and SNAP (10 *μ*M) in the presence of different formulations of GO, PEI, PEG, and PEI-PEG@GO at the concentration of 250 *μ*/ml (n = 3 independent samples).

To investigate the real-time kinetics of NO release from *S*-nitrosothiols (RSNO) we used a NO electrochemical sensor in phosphate-buffered saline (PBS) (pH 7.4) at 37 °C under continuous stirring, which can selectively detect the generation of NO concentrations above ≈1nM in real-time.^47^ EDTA was used asa metal ion ligand to block the spontaneous decomposition of both NO sources in the presence of impurities.^48^ *S*-nitroso-N-acetylpenicillamine (SNAP) was chosen as a model RSNO, and *S*-nitrosoglutathione (GSNO) was chosen as one of the most abundant endogenous NO donors, present ranging from 0.02 to 0.2 *μ*M in human blood.^49,50^ GSNO or SNAP (10 *μ*M) was added to the PBS solution with or without different compounds to detect NO generation over time. GSNO and SNAP alone were found to spontaneously produce NO up to 19.5 and 11.5 nM/min, respectively. However, the use of PEI-PEG@GO with GSNO or SNAP synergistically increased the rate and quantity of NO production compared to any component alone (Fig. 3). We postulate that the fast rate of NO generation was facilitated by the high density of amine groups on the surface of GO, while the slow rate of NO generation after 20 min (GSNO) and 35 min (SNAP) was facilitated by the amine groups present within the layers of GO. These results demonstrate the possibility of using functionalized GO to catalytically generate NO from endogenous NO sources by tailoring the form of polymers.

**FIG. 4:**
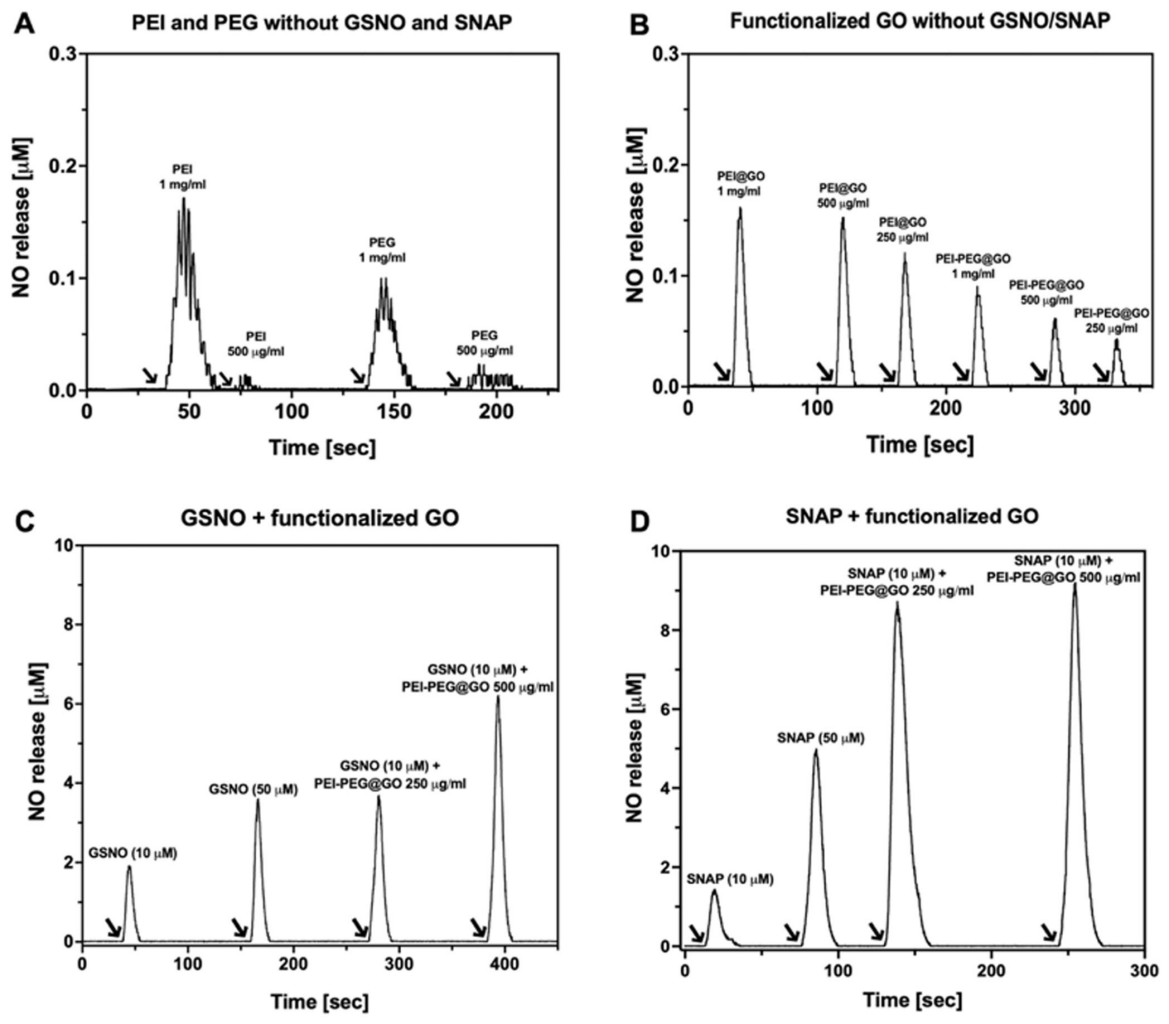
Catalytic generation of nitric oxide (NO) from GSNO and SNAP using PEI, PEG, and PEI-PEG@GO at different concentrations in PBS buffer (pH 7.4) in the presence of EDTA as determined by a chemiluminescence NO analyzer. (a) Representative NO release profile from PEI and PEG without GSNO and SNAP. (b) Representative NO release profile from PEI@GO and PEI-PEG@GO without GSNO and SNAP. (c) Representative NO release profile from GSNO and PEI-PEG@GO+GSNO. (d) Representative NO release profile from SNAP and PEI-PEG@GO+GSNO. The arrow shows the injection of compounds. Time on the x axis represents the time since the start of the chemiluminescence time-trace.

The total capacity for NO production was then measured by ozone-based chemiluminescence using either GSNO or SNAP as substrates. In this assay, the conditions promote rapid decomposition of NO, producing a single narrow peak calibrated for NO concentration. Panels A and B in Fig. 4 demonstrate that in the absence of the substrate, there is negligible NO production from PEG or PEI alone or for any formulation of functionalized GO [note y axis scale relative to panels (c) and (d)]. Spontaneous NO release was observed with GSNO alone, but total NO production was much higher in the presence of PEI-PEG@GO and in a concentration-dependent manner, which is consistent with a catalytic effect of PEI-PEG-GO [Fig. 4(c)]. A similar pattern was observed when using SNAP as a substrate, but with even higher levels of NO production [Fig. 4(d)]. PEI-PEG@GO was calculated to release up to 62% of the available NO from GSNO and 91% from SNAP, indicating a high reaction efficiency. Collectively, these results demonstrate that controlled NO generation can be achieved by tuning the concentrations of PEI-PEG@GO and GSNO or SNAP.

### Coating of functionalized graphene on 3D-printed stent material

**FIG. 5:**
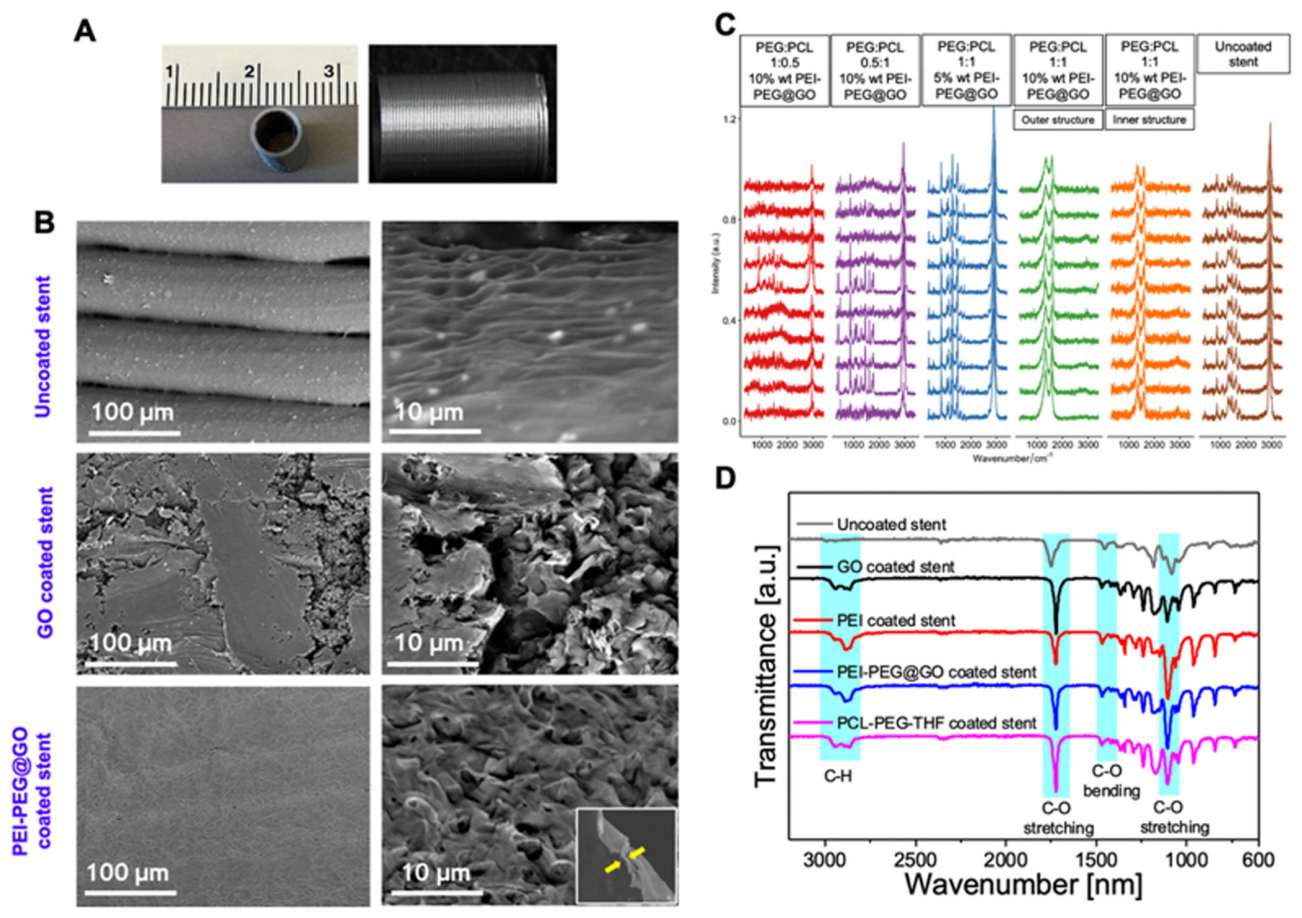
Characterization of the coating of PEI-PEG@GO on the stent material (PLA). (a) Photographs of 3D-printed stents. (b) SEM micrographs of coated and uncoated stents at different magnifications. The inset figure in PEI-PEG@GO coated stent shows the average thickness of coating which is 0.2 *μ*m. (c) Raman spectra of uncoated and coated stents (with PEI-PEG@GO) using different combinations of coating solutions and PEI-PEG@GO. Three random fields of view were chosen with ~18 random points mapping per field view along with ~54 points per stent. The optimized coating solution (PEG:PCL 1:1 and 10 wt. % PEI-PEG@GO) was prepared after screening different ratios of PEG and PCL in THF with PEI-PEG@GO. The weight percentage of PEG, PCL, and PEI-PEG@GO in the coating solution is 47.5%, 47.5%, and 5%, respectively. Each spectrum in this panel represents characterization at a spatially distinct sampling point. (d) FTIR spectra of uncoated and coated stents under different conditions. The characteristic FTIR bands are shown along with region names.

One objective of this work was to use a simple and easy-to-apply coating method to provide a uniform layer on both the outer and inner shells of the biodegradable stent material. 3D-printed polylactic acid (PLA) stent material of 4 mm inner diameter, 5 mm outer diameter, and 8 mm length exhibited well-defined edges [Fig. 5(a)]. SEM micrographs showed that the uncoated stent had a well-defined geometry with a smooth surface finish, while GO and PEI-PEG@GO coatings had a layered-like morphology [Fig. 5(b)]. The average thickness of coating is 0.2 *μ*m. The coating roughness was measured by the difference in height between the highest and lowest coated surface as 0.2 ± 0.01 *μ*m. The 3D-printed stent material was dip-coated by immersing in a solution of tetrahydrofuran (THF), PCL, and PEG. THF was used as a solvent since it evaporates rapidly, leading to the formation of a uniform surface layer; a combination of PCL and PEG was used to increase the viscosity and reduce the surface tension of the coating solution to avoid the likelihood that THF would dissolve the PLA-based stent. The coating solution was optimized to achieve the highest coating uniformity by varying the ratios of PEG to PCL and using Raman spectroscopy to assess uniformity at multiple points on the stent surface [Fig. 5(c)]. A 1:1 ratio of PEG and PCL at 10% by weight of GO provided the most uniform coating as demonstrated by the distinct Raman D (1350 cm^−1^) and G (1600 cm^−1^) peaks characteristic of GO.^44^ The absence of peaks that are typical for PLA, suggests that PEI-PEG@GO successfully coated the stent both inside and out. Unsuccessful coating formulations had Raman spectra similar to the uncoated stent material with PLA-associated peaks at 2992, 1752, 1457, and 876 cm^−1^.^51^

Similarly, FTIR spectra suggested complete coating and cross-linking of the stent material, as evidenced by the peaks at 2800, 1750, and 1100 cm^−1^ [Fig. 5(d)]. The FTIR spectrum of PLA shows a peak at 1100 cm^−1^ that corresponds to the vibrations of the C=O bond, and a peak at 1750 cm^−1^ that corresponds to the stretching vibrations of the C=O bond; in the case of coated PLA, the new band at 2800 cm^−1^ corresponds to the asymmetrical and symmetrical stretching vibrations of the C-H. The spectra of the coated PLA are similar to the PLA spectrum, except for the peak of PEI-PEG@GO, suggesting the coverage of PLA by functionalized GO.

### Hydrolytic degradation testing of coated and uncoated stents

**FIG. 6:**
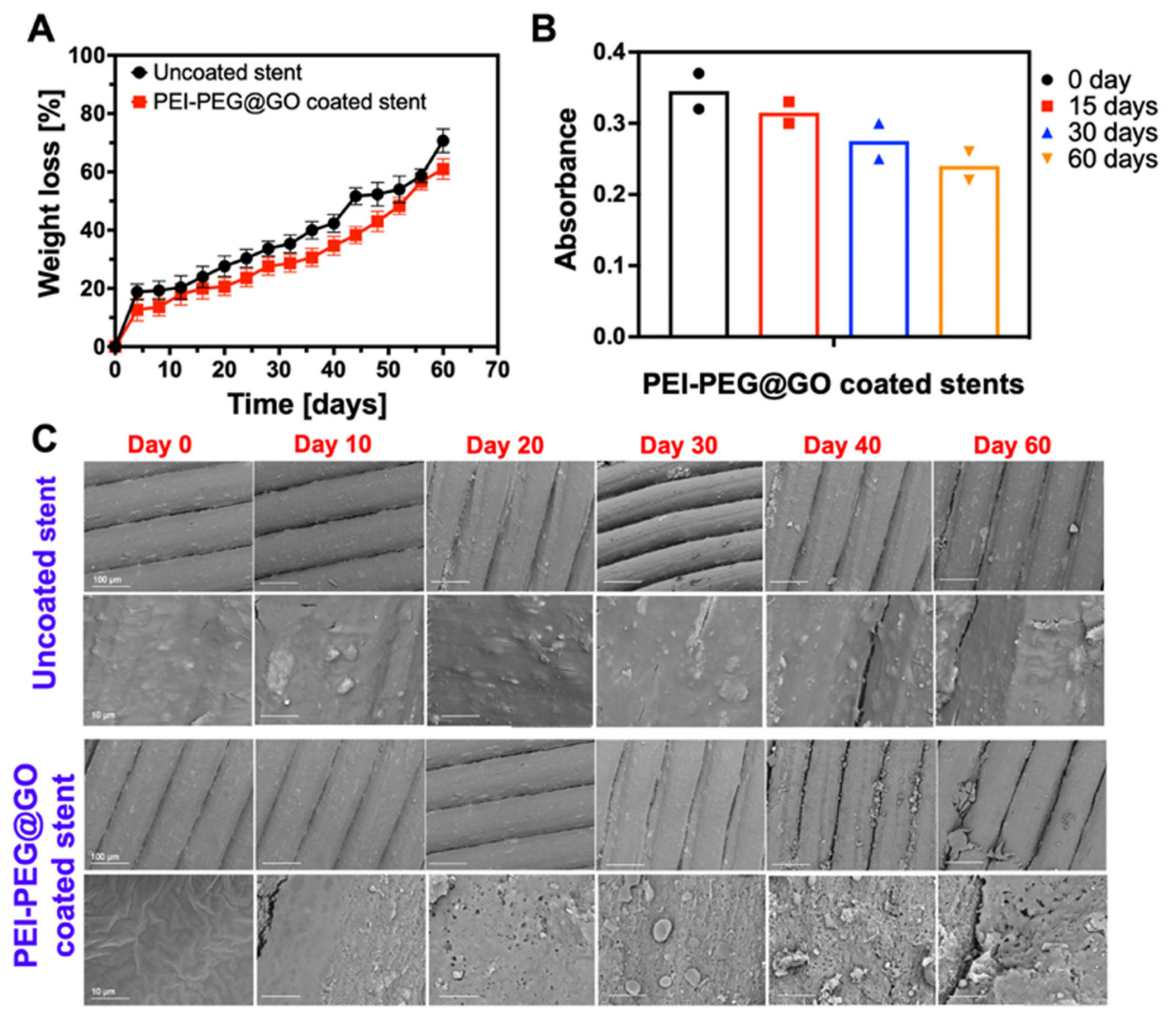
Accelerated hydrolytic degradation of coated and uncoated stent material at elevated temperature. (a) Weight loss of stents as a function of time in PBS (pH 7.4) at 60 °C for 60 days. The data are shown as the mean ± SD, *n* = 3. (b) The stability of amine groups on stents over 60 days under hydrolytic degradation conditions is presented in (a). The stability of amine groups was measured using a standard colorimetric assay based on Orange acid II (OA II). The data are shown as the mean of two independent experiments. (c) SEM micrographs of the surface of the stent material at different time points of the hydrolytic degradation experiments at 60 °C in PBS (pH 7.4).

The hydrolytic degradation of polymers under physiological conditions is slow, taking months to several years;^52^ therefore, accelerated degradation at higher temperatures is widely used to study the decomposition of materials over shorter timescales.^52,53^ The hydrolysis of PLA will result in the release of lactate monomers from the polymer structure, which will be either excreted or metabolized *in vivo*. However, it was unknown how the degradation rate of PLA will be altered by the presence of a PEI-PEG@GO coating. We, therefore, measured degradation rates of coated and uncoated stents at 60 °C to provide accelerated hydrolytic conditions.^54^ In the case of uncoated PLA, the weight loss was 20% by day 4, and reached 72.5% by day 60, while weight loss for PEI-PEG@GO coated PLA was slightly delayed, reaching 17% by day 12 and 61% over 60 days [Fig. 6(a)]. At day 56, the degradation curve of both coated and uncoated materials was observed to converge; however, the coating remained mostly intact as indicated by the continued presence of amine groups measured by colorimetric assay [Fig. 6(b)]. The stability of amine groups depends on the nature and strength of bonding,^55^ and the amine content will typically decrease at higher temperatures.^56^ Their presence after two months is likely due to the strong intermolecular interaction between GO and amine groups via *π–π* bonding. The morphology of degraded stents at each time point was evaluated using SEM to further explore the degradation pathways [Fig. 6(c)]. The stent design was preserved under all conditions and time points, demonstrating that this is a degradation process rather than due to erosion. Taken together, it can be concluded that the PEI-PEG@GO coating alters the degradation kinetics of PLA-based stents, but there was convergence at longer time points, suggesting that the overall life span of the stent was not significantly altered. Given the exposure of coronary stents to mechanical stress, coating adherence is critically important for the safe and effective use of stents *in vivo*, it is therefore notable that the PEI-PEG@GO coating remained largely intact over the period tested.

### Stability testing of coated stent materials

**FIG. 7:**
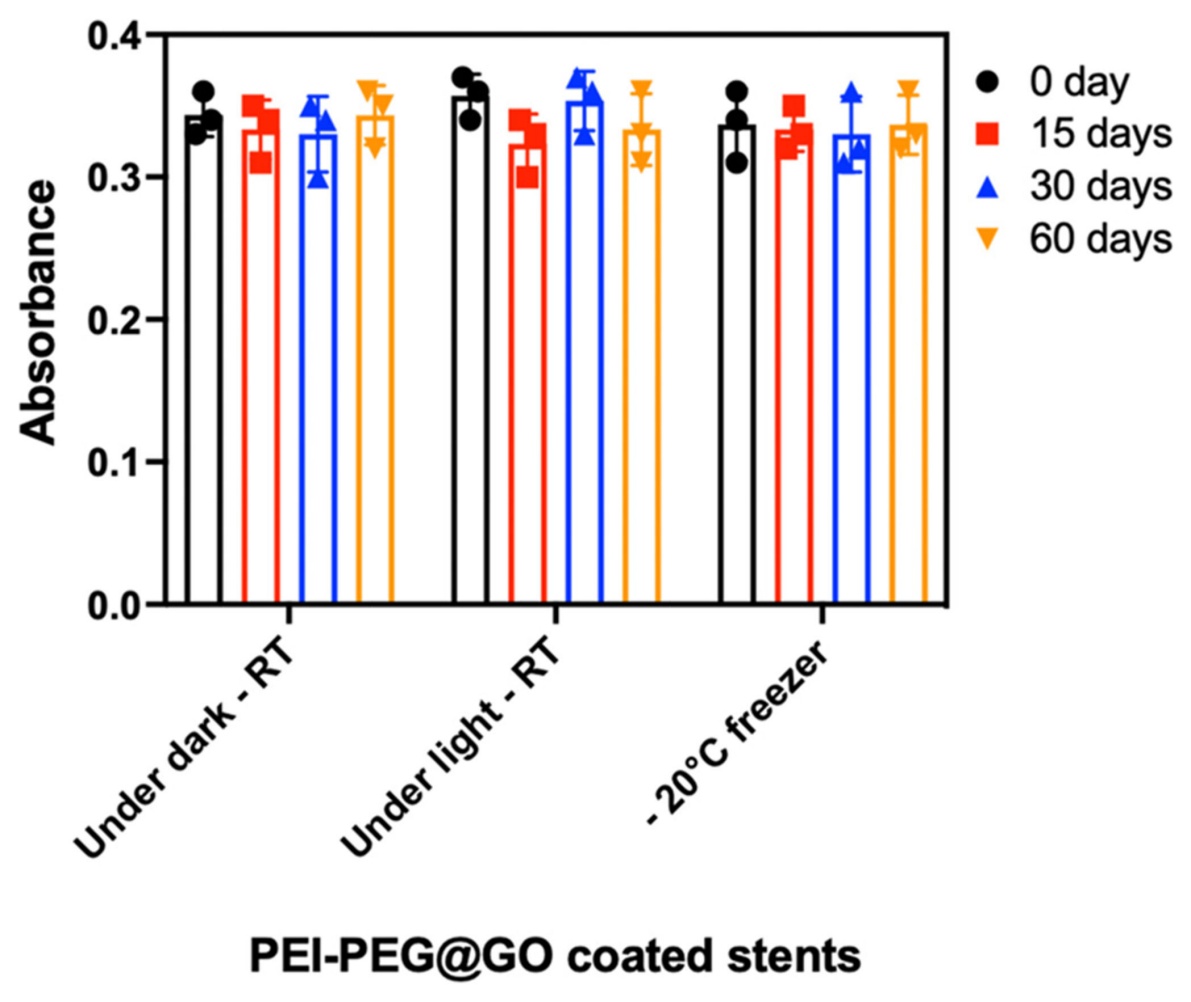
The stability of amine groups on stents for time points up to 60 days. The data are shown as mean ± SD, *n* = 3. One-way ANOVA test was used to calculate statistical significance. RT, room temperature.

Graphene nanostructures remain remarkably chemically stable under ambient conditions,^57^ and it has been shown previously that coupling of PEG improves the stability of PEI.^42^ To investigate the stability of the PEI-PEG@GO coating, we evaluated the stability of the conjugated amine content on 3D-printed PLA under different conditions (dark at room temperature, light at room temperature, and dark at −20 °C) using a standard colorimetric assay based on Orange acid II (OA II).^58,59^ No detectable difference was observed between day 0 and day 60 under any of the tested conditions, indicating that the amine content was highly stable (Fig. 7).

The functionalization of GO with two amine-rich polymers (i.e., PEI and PEG) lays a foundation for the development of novel NO-generating coatings for biomedical applications. However, extensive studies are required to develop this concept further, not least, to explore how these materials interact with biological environments. In the first instance, this will require *in vitro* testing to demonstrate the survival, adhesion, and proliferation of endothelial and smooth muscle cells when seeded onto scaffolds coated with PEI-PEG@GO and to explore the effect on platelets when exposed to whole blood. The stability of coating adherence and degradation kinetics will need to be tested under conditions of mechanical stress, such as those experienced during implantation, and also under conditions that mimic the shear stress of blood flow when the device is *in situ*. Coatings on medical devices can also degrade over time due to physiological interactions with cells, circulating macromolecules, and enzymatic activity, so these aspects also need to be tested *in vivo* by implantation of coated devices in animal models to assess the fate, elimination, and potential toxicity of degradation products. It has previously been shown that the degradation of GO can release small carbon fragments.^60^ Furthermore, PEG functionalized GO did not accumulate in the lung or induce pulmonary toxicity, which is a major issue for non-functionalized carbon nanomaterials.^61^ However, the physiological impact is dependent on size, concentration, and the presence of functional groups, which means that all new formulations are unique and require specific testing for toxicity and mechanisms of degradation. However, it should be noted that the total quantity of coating on a stent is relatively small and will degrade over a prolonged period, such that acute toxicity and accumulation are unlikely. Ultimately, *in vivo* efficacy needs to be demonstrated in animal models of vascular injury with postmortem histopathology to evaluate thrombosis, inflammatory responses, re-endothelialization, neointimal hyperplasia, and restenosis.

## Conclusion

In this experimental study, we integrated multiple desirable functions into one vascular stent through 3D printing and amine-catalyzed NO generation. The use of amine-functionalized graphene oxide (GO) as a NO catalyst is an important step toward engineering NO-releasing cardiovascular implants with extended delivery times. The NO generated from functionalized graphene was 0.7 × 10^−10^ and 1.1 × 10^−10^ mol cm^−2^ min^−1^ from 10 *μ*M GSNO and 10 *μ*M SNAP, respectively. Therefore, NO generation from functionalized graphene is indeed physiologically relevant to the healthy endothelium, which continuously produces NO at the rate of 0.5–4 × 10^−10^ mol cm^−2^ min^−1^. Although the NO generation capacity of the graphene-coated stent will depend on the levels of GSNO and RSNO present in blood vessels. In comparison to exogenous NO donors, NO-generating nanomaterials show promise to overcome the inherent limited loading capacities of NO donors and associated toxicity. This is the first report showing that amine-functionalized GO can catalytically decompose GSNO and SNAP to generate physiologically significant levels of NO. The underlying mechanism of the catalytic role of amine-functionalized GO involves the nucleophilic reaction between the primary amines of PEI on GO and the SNO groups of GSNO and SNAP. These results demonstrate that amine-functionalized GO maintains its structural integrity under physiologically relevant conditions, which is of paramount importance to avoid aggregation/agglomeration and reduce its toxicity. Taken together, our results provide new opportunities for the development of novel materials for endogenously generating NO.

## Materials and Methods

### Chemicals and reagents

Sodium nitrate (NaNO_3_), sulfuric acid (H_2_SO_4_ - 95.0%–98.0%), potassium permanganate (KMnO_4_), hydrogen peroxide (H_2_O_2_ - 30 wt. %), and hydrochloric acid (HCl - 36 wt. %) were purchased from Thermo Scientific, Fisher Scientific, Acros, Nacalai Tesque, and Alfa Aesar, respectively. Graphite flakes, branched PEI with molecular weight (MW) of 25kDa, *N*-(3-dimethylaminopropyl-*N*′-ethylcarbodiimide) hydrochloride (EDC), 6-armed amine-terminated polyethylene glycol (PEG) with MW of 10 kDa, phosphate-buffered saline (PBS), sodium nitrite (NaNO_2_, 97%), potassium iodide, vanadium chloride, *S*- nitroso-*N*-Acetyl-D,L-penicillamine (SNAP), and *S*-sitroso-L-glutathione (GSNO), polycaprolactone (PCL, Mn = 80 000), tetrahydrofuran (THF), EDTA, TEM copper grid, and Orange acid II (OA II) were purchased from Sigma Aldrich. All materials were used as received without any further purification unless stated otherwise.

### Synthesis of graphene oxide

As we previously reported, exfoliated graphite was used to create exfoliated graphene oxide (GO) flakes using a modified Hummer’s method.^34,35,44^ An 800 ml round-bottom flask containing 2 g of graphite flakes was filled with 1.5 g of NaNO_3_ and 150ml (98%) H_2_SO_4_.The reaction mixture was mixed using magnetic stirring, and the flask was then submerged in an oil bath. The mixture was then heated to 35 °C prior to the addition of KMnO_4_ (9 g). The solution was stirred for 24 h, after which 280 ml of H_2_SO_4_ (5%) was added, the temperature raised to 85–95 °C, and stirred for an additional 2 h before being allowed to cool to 60 °C. Subsequently, a solution of 15 ml of H_2_O_2_ (30 wt. %) was introduced, followed by a further 2 h of stirring. To eliminate impurities, the product was washed 7–8 times with HCl (3 wt. %) and 4–5 times with distilled water. As prepared GO was dissolved in water for further processing.

### Synthesis of functionalized graphene oxide

GO was functionalized with PEI and PEG by forming an amide bond between PEI, PEG, and GO in the presence of EDC. To synthesize PEI-PEG@GO, GO (10 mg) was dispersed in distilled water (10 ml) and was then mixed with 5 ml of 6-armed amine-terminated PEG at the concentration of 0.5 mg/mL in water The mixture was sonicated for 5 min. 5 ml of EDC at the concentration of 2 mg/mL was added to the reaction solution and was sonicated for another 5 min. The solution was stirred at room temperature for 10 min. The solution was sonicated for 5 min following the addition of 10 ml of PEI (5 mg/mL). 20 ml of EDC was added again (0.5 mg/mL) and was stirred at room temperature overnight. The product was washed according to the following: the resultant product was dissolved in NaCl (1.6 g) and centrifuged at 13 000 rpm for 2 h and subsequent filtration using a 100 K ultrafilter. After ultrafiltration, the product was washed three to four times with an aqueous solution containing 10% NaCl to eliminate any remaining PEI, PEG, and urea. Free PEI and PEG in the ultrafiltrate were detected by Orange acid II (OA II), to confirm the complete removal of unreacted PEI and PEG. The step-by-step protocol of OAII is given in the section on stability testing. After removing NaCl from the product by multiple washes with distilled water, it was dispersed in water at required concentrations. To synthesize PEI@GO, the same procedure was followed except without the addition of PEG.

### Physical and chemical characterization of functionalized graphene oxide

A drop of the graphene samples was placed on holey carbon copper grids for the examination of their morphology using transmission electron microscopy (TEM). (Protochips, Morrisville, NC, USA). Images were obtained using a 300 kV Titan Krios electron microscope that was equipped with a Falcon 3 detector (Thermo Scientific, Waltham, MA, USA) and a Cs corrector (CEOS). Cu K*α* radiation was used for x-ray diffraction (XRD) investigation; 40 kV of voltage and 40 mA of current were used during the tests. The spectra were obtained with a step time of 1 s and a step size of 0.02° at 2*θ*. Fourier transform infrared (FTIR) spectroscopy measurements were obtained using a Tensor-27 FTIR spectrometer (Bruker Optics, Champs-sur-Marne, France) within the 4000–500 cm^−1^ wavenumber range. Samples were mixed with potassium bromide (KBr) to collect FTIR spectra. Raman spectroscopy measurements were performed using laser excitation at 532 nm (Renishaw, Stroud, UK). A synthetic air environment was used for TGA experiments using a TA Instruments Q5000, with a heating ramp of 10 °C/min. A contact angle goniometer was used to calculate the water solubility of GO, PEI@GO, and PEI-PEG@GO. The photos were taken with a digital camera, and the contact angle was calculated with ImageJ software. A 10 *μ*L drop was placed onto a glass slide to create the contact angle surfaces.

### Measurement of NO generation

The NO generated from the samples GO, PEI, PEG, PEI-PEG@GO, and PEI@GO with and without GSNO and SNAP was purged from the test solution using helium gas and detected with a chemiluminescence NO analyzer (NOA) (Seivers 280, Boulder, CO).^62,63^ PBS (pH 7.3) comprising of 0.5 mM EDTA was used as the working solution in order to prepare the required concentrations of GSNO and SNAP. A 10 *μ*l aliquot of each sample was added into the purge vessel (with a constant flow/purge of helium gas) using a gas-tight syringe through the injection unit of the vessel, The purging vessel was covered with aluminum foil in order to prevent the light exposure. Each measurement was continued until the NO spectrum returned to the baseline levels within several minutes. The calibration curve was obtained via sodium nitrite reduction in an acidified vanadium chloride solution. The amount of NO generated from the sample was determined from the calibration curves. Microsoft Excel 2010 (Microsoft, Redmond, WA, USA) was used to process raw data.

A free radical analyzer (TBR4100, World Precision Instruments) fitted with a NO-sensitive electrode (ISO-NOP, World Precision Instruments) was used to investigate real-time NO production. The desired concentrations of GSNO and SNAP were prepared in PBS solution (pH 7.3) containing 0.5 mM EDTA. The reference electrode was polarized and calibrated according to the manufacturer’s instructions. The probe was submerged in 10 ml 0.1 M H_2_SO_4_/0.1 M potassium iodide solution in a glass vial in order to achieve a stable and flat current baseline. To produce NO concentrations for the calibration curve, the mixture was subjected to increasing amounts of 25 *μ*M sodium nitrite solution. Since the ratio of sodium nitrite to NO was stoichiometric (1:1), the amount of sodium nitrite input was used to calculate the generated NO concentrations. To detect the concentration of NO produced from GSNO by PEI, PEG, GO, and PEI-PEG@GO, the NO probe was submerged in a glass vial that was filled with 4 ml of PEI, PEG, GO, and PEI-PEG@GO (125–1000 *μ*g mL^−1^) in PBS buffer (pH 7.3). Once a steady flat baseline was reached, 50 *μ*L of GSNO solution was added to the vial, bringing the final concentration to 10 *μ*M. The generated NO was examined from the calibration curve, and changes in the current response were recorded in real time. Glass vials were shielded by aluminum foil in order to protect the sample solutions from light exposure and maintained at 37 °C using a magnetic stirrer plate with constant stirring.

### 3D printing of coronary artery stent materials

The computer-aided design (CAD) of the stent was developed using Blender software (Blender^®^ 2.79b – Open-source 3D editor); Ray Ware, a program, was used to print it (Ray Ware 1.4.5). An STL file was generated and tested for errors and modified according to the required dimensions. In order to slice the 3D model into multiple thin layers and to generate the coordinates (G-code), the Standard Triangle language (STL) file was processed using a slicer software A Prusa MK3S 3D printer containing a 0.25 mm nozzle and Prusament PLA filament was used to prepare the stent. The nozzle and bed temperatures were set to 215 and 65 °C. The layer height was set to 0.1 mm to achieve high-quality and accurate prints. Stents of 4 mm inner diameter, 5 mm out diameter, and 8 mm length were printed.

### Preparation of the graphene coating on stent materials

In order to prepare the coating solution, 100 mg of PEG and PCL (1:1) were dissolved in 1 ml of THF with continuous stirring at room temperature for 90 min. After adding 10wt. % of pristine and functionalized GO to the mixture, it was stirred for an additional 60 min to ensure that all of the materials were dissolved. Stents were dipped in the coating solution for a few seconds and then kept in the dark at room temperature for solvent evaporation. The stents were vacuum-dried and stored at −20 °C.

### Characterization of the coated stent materials

The morphologies of the materials were assessed using a JEOL JSM-7500F field-emission scanning electron microscope (SEM) with 50 mm^2^ X-MAX detector from Oxford Instruments (Abingdon, United Kingdom). The coated and uncoated materials were cut using a sharp blade. The specimens were sputter-coated with gold. To measure the thickness of the coatings, the coated stent material was cut and mounted vertically on the sample holder. The SEM micrographs were recorded at the cross section of the coated PLA stent. The data were analyzed by ImageJ software (NIH, Version). The samples were also characterized using FTIR. The details of the instrument have been given in the section titled Physical and chemical characterisation of functionalized graphene oxide. FTIR samples were prepared by cutting the samples using a sharp blade. Raman spectra were obtained using a confocal Raman microscope (LabRAM HR Evolution, Horiba Scientific, UK) equipped with a motorized XYZ stage, an integrated microscope (BX41, Olympus), and a 532-nm laser. Spectra were collected using a 50× air objective lens (numerical aperture 0.5), a 0.5-s acquisition time, and a 300 grooves/millimeter diffraction grating for an input laser power of 2–4 mW passing through a 25% neutral density (ND) filter. In each condition, 100–150 spectra were acquired with a spatial resolution at ~1.5 *μ*m between each spectrum. The random fields of view were selected for each sample with ~18 random points mapping per field view and ~54 points per sample.

### Biodegradability testing of the coated and uncoated stent materials

Stents were weighed and kept at 60 ± 1 °C in individual vials containing 5 ml of PBS at pH 7.4 (n = 3/group). The stent was cleaned with distilled water at room temperature every four days, and it was vacuum-dried at room temperature until a consistent weight was achieved. The dried samples were weighed to determine the average mass loss at each time point (equation below). The PBS solution was changed weekly. The surface of the coated and uncoated stents at the end of each experiment was analyzed using SEM.

Weight loss(%)=100×(Final weight−initial weight)/initial weight

### Stability testing of the coated stent materials

The stability of amine groups and shelf-storage stability of coated stents were performed by the quantification of amine groups following the methods reported.^59,64^ To determine the surface density of amino groups, the samples were first immersed in Orange acid II (OA II) solution (500 mM in HCl solution; pH 3). Weakly attached OA II was eliminated from the samples by washing them with HCl solution (pH 3) following a 12 h incubation period at 37 °C. The samples were then dissolved for 15 min at 37 °C in a NaOH solution (pH 12) to free the electrostatically bound OA. Using a microplate reader with background subtraction at 485 nm, the OA concentration corresponding to the density of amine groups on the samples was determined colorimetrically. The samples were tested under different conditions, including under dark stored at −20 °C, under dark stored at room temperature, and under light stored at room temperature.

## Statistical analysis

The NO generation data presented as the mean ± standard deviation were collected in triplicate (n = 3). The standard deviation measurements are represented by error bars for the hydrolytic degradation (n = 4), and shelf-storage stability (n = 3). The sample size has also been given in figure legends. The statistical significance was determined using a one-way ANOVA test.

## Data Availability

The data that support the findings of this study are available from the corresponding author upon reasonable request.

## Significance Statement

Coronary artery disease is the number one leading cause of death in Europe and North America. It arises due to a narrowing of the blood vessels that supply the heart itself, thereby causing angina and heart attacks. Narrowed arteries are commonly treated by the insertion of a stent, which is a tube of expandable metal mesh that physically keeps the artery open. Stents were originally made from bare metal, but these often caused re-narrowing of the vessel (restenosis), and most are now coated to release drugs to prevent this (drug-eluting stents). However, these can delay endothelial cell revascularization and are associated with late blood clotting (thrombosis). Nitric oxide (NO) is continually generated in healthy blood vessels, where it is critically important in maintaining vessel integrity, so an ideal stent would mimic the properties of NO. Herein, we report for the first time on an amine-functionalized graphene coating for bioresorbable stents, which generates NO from *S*-nitrosothiols that are typically found in the bloodstream. This represents a new class of stent coating, which is easy to produce and can be tuned to release NO over prolonged periods of time by using endogenous substrates. Ultimately, this has the potential to change clinical practice and the basic principle is also applicable to a wide range of medical implants.
